# Chlorogenic Acid and Quercetin in a Diet with Fermentable Fiber Influence Multiple Processes Involved in DSS-Induced Ulcerative Colitis but Do Not Reduce Injury

**DOI:** 10.3390/nu14183706

**Published:** 2022-09-08

**Authors:** Leigh Ann Maslin, Bradley R. Weeks, Raymond J. Carroll, David H. Byrne, Nancy D. Turner

**Affiliations:** 1 Intercollegiate Faculty of Nutrition, Texas A&M University, College Station, TX 77845, USA; 2 Department of Veterinary Pathobiology, Texas A&M University, College Station, TX 77845, USA; 3 Statistics Department, Texas A&M University, College Station, TX 77845, USA; 4 Department of Horticultural Science, Texas A&M University, College Station, TX 77845, USA; 5 Department of Food Science & Human Nutrition, Michigan State University, East Lansing, MI 48824, USA

**Keywords:** ulcerative colitis, pectin, quercetin, chlorogenic acid, inflammation, gene expression

## Abstract

Ulcerative colitis (UC) patients often avoid foods containing fermentable fibers as some can promote symptoms during active disease. Pectin has been identified as a more protective fermentable fiber, but little has been done to determine the interaction between pectin and bioactive compounds present in foods containing that fiber type. Quercetin and chlorogenic acid, two bioactives in stone fruits, may have anti-cancer, anti-oxidant, and anti-inflammatory properties. We hypothesized that quercetin and chlorogenic acid, in the presence of the fermentable fiber pectin, may suppress the expression of pro-inflammatory molecules, alter the luminal environment, and alter colonocyte proliferation, thereby protecting against recurring bouts of UC. Rats (*n* = 63) received one of three purified diets (control, 0.45% quercetin, 0.05% chlorogenic acid) containing 6% pectin for 3 weeks before exposure to dextran sodium sulfate (DSS, 3% for 48 h, 3x, 2 wk separation, *n* = 11/diet) in drinking water to initiate UC, or control (no DSS, *n* = 10/diet) treatments prior to termination at 9 weeks. DSS increased the fecal moisture content (*p* < 0.05) and SCFA concentrations (acetate, *p* < 0.05; butyrate, *p* < 0.05). Quercetin and chlorogenic acid diets maintained SLC5A8 (SCFA transporter) mRNA levels in DSS-treated rats at levels similar to those not exposed to DSS. DSS increased injury (*p* < 0.0001) and inflammation (*p* < 0.01) scores, with no differences noted due to diet. Compared to the control diet, chlorogenic acid decreased NF-κB activity in DSS-treated rats (*p* < 0.05). Quercetin and chlorogenic acid may contribute to the healthy regulation of NF-κB activation (via mRNA expression of IκΒα, Tollip, and IL-1). Quercetin enhanced injury-repair molecule FGF-2 expression (*p* < 0.01), but neither diet nor DSS treatment altered proliferation. Although quercetin and chlorogenic acid did not protect against overt indicators of injury and inflammation, or fecal SCFA concentrations, compared to the control diet, their influence on the expression of injury repair molecules, pro-inflammatory cytokines, SCFA transport proteins, and NF-κB inhibitory molecules suggests beneficial influences on major pathways involved in DSS-induced UC. Therefore, in healthy individuals or during periods of remission, quercetin and chlorogenic acid may promote a healthier colon, and may suppress some of the signaling involved in inflammation promotion during active disease.

## 1. Introduction

Inflammatory bowel disease (IBD) is one of the top five digestive diseases in the United States, with ulcerative colitis (UC) being the most prevalent form [1,2]. Inflammation in UC is localized to the distal colon, and, because colon cancer is more prevalent in the distal colon and rectum, patients with recurrent UC are at a particularly high risk of developing colon cancer [2,3,4]. The primary causes of UC are not known, yet the disease is associated with exaggerated immune responses to normal commensal microbiota, a probable dysbiosis in which the distribution of microflora is altered, and epithelial barrier function is impaired [5]. Treatment regimens relying upon pharmaceuticals and biological therapies are currently only effective in approximately half of subjects [6]. Therefore, by identifying dietary modifications that either suppress inflammation in the colon, or mediate the contributors to colitis or their downstream effects, the morbidity associated with UC may be reduced along with the incidence of colon cancer.

Diets rich in fruits and vegetables are associated with a reduced risk of many diseases, including colon cancer and IBD [7,8,9,10]. Fruits and vegetables are good sources of fiber, vitamins, and minerals, as well as many different types of phytochemicals. Stone fruits, such as peaches and plums, contain mostly fermentable fibers like pectin with biologically active phytochemicals, including quercetin and chlorogenic acid [11,12,13]. The most abundant flavanol in the human diet is quercetin, and chlorogenic acid, an ester of caffeic acid, is the most abundant hydroxycinnamic acid [11,13,14]. A limited number of studies have attempted to determine the effect of stone fruits on colon health, with most of the work being conducted in vitro. Yang and Gallaher [15] found that inclusion of prunes in the diet of rats altered the activities of bacterial enzymes leading to a reduction in fecal levels of secondary bile acids, which are known irritants that can promote colon carcinogenesis [15]. Phenolic extracts of Kakadu plums have been reported to decrease activation of NF-κB and cyclooxygenase (COX)-2 levels in LPS-stimulated murine macrophages [16]. Since stone fruit extracts contain many different compounds that could be conferring those benefits, it is desirable to identify the effect of individual compounds within the complex mixture present in the whole fruit.

In an attempt to understand how an individual compound influences the colon, our earlier work demonstrated that quercetin reduces colon carcinogenesis in vivo, and that the suppression may have occurred in part due to a reduction in expression of pro-inflammatory mediators like COX-2 [17]. Our results complement prior studies, which showed that quercetin suppresses activation of NF-κB in an acute model of colitis, and enhances epithelial barrier function in vitro by increasing expression of the gene encoding claudin-4, a tight junction protein [18,19]. In UC, epithelial barrier integrity is reduced, resulting in increased paracellular transport of luminal contents, triggering immune and inflammatory responses [5]. The effects of quercetin on pro-inflammatory cytokines and epithelial integrity have not been studied in chronic colitis.

Chlorogenic acid also may protect against inflammation in the colon and inflammation-associated colon carcinogenesis. Chlorogenic acid has been shown to inhibit DNA damage and the formation of mutagenic and carcinogenic compounds in vitro [14]. Esters of caffeic acid also inhibit pro-tumorigenic lipoxygenase and cyclooxygenase in cell culture [20]. Few studies have assessed the anti-inflammatory effects of chlorogenic acid in vivo, but hydrocaffeic acid, a derivative of caffeic acid, has been shown to alleviate DSS-induced increases in IL-1, TNF-α, and IL-8 in acute colitis [21]. The anti-cancer and anti-inflammatory properties demonstrated in vitro and in vivo warrant further investigation of chlorogenic acid in an in vivo model of chronic colitis.

Dietary components including dietary fiber and bioactive compounds, such as quercetin and chlorogenic acid, influence colonic microbiota [22,23,24,25]. Chlorogenic acid and quercetin are metabolized by bacterial enzymes and, therefore, may influence microbial fermentation patterns [26]. Changes in the microbial populations and their fermentation patterns could be among the mechanisms whereby these bioactive compounds impact the colonic epithelium and the production of pro-inflammatory cytokines. Most of the previously cited work using stone fruits, their isolates, or individual compounds has been conducted using pelleted diets that include cereal grains containing fibers with low fermentability, or with purified diets containing cellulose. It is therefore not clear how quercetin and chlorogenic acid may influence disease processes in the presence of fermentable fibers such as pectin.

The purpose of this study was to evaluate the effects of quercetin and chlorogenic acid in animals consuming the fermentable fiber pectin on colon inflammation and injury, as well as the colon luminal environment using a model of chronic colitis. To determine the mechanisms involved, the expression of genes for proteins involved in the NF-κB, toll-like receptor (TLR), and tumor necrosis factor receptor (TNFR) pathways were examined. Based on the literature, we hypothesized that the stone fruit bioactive compounds quercetin and chlorogenic acid may protect against inflammation and injury in a chronic colitis model by suppressing the expression of pro-inflammatory mediators, and by altering the cell cycle and luminal environment.

## 2. Materials and Methods

### 2.1. Animals, Diets and Study Design

Animals in this project were treated in accordance with the National Institutes of Health guidelines using a protocol approved by the Texas A&M University Animal Care Committee (AUP #2008-114). Sixty-three male weanling Sprague-Dawley rats (Harlan Sprague-Dawley, Houston, TX, USA) were stratified by body weight and assigned to one of three diet groups (basal, 0.45% quercetin, 0.05% chlorogenic acid, Sigma Aldrich, St. Louis, MO, USA), which were further divided into dextran sodium sulfate (DSS, 3%, 36,000–50,000 MW; MP Biomedicals, Irvine, CA, USA) and control (0% DSS) groups (Supplemental Table S1). This level of quercetin has been shown to inhibit expression of COX-1 and COX-2 in an AOM model [17] and this level of chlorogenic acid is similar to that obtained through drinking coffee and consuming stone fruits [13,27,28]. All diets contained 6% pectin as the fiber source to represent the predominant fiber type found in stone fruits.

After 3 wks of consuming the experimental diets, DSS treatments were initiated, which included three 48-h treatments (3% DSS in H_2_O; d 22, 34, 48), each separated by a 2 wk recovery period [29]. Control animals received only distilled H_2_O, as did the DSS rats between DSS treatment periods. DSS induces colitis with many of the clinical and histopathological characteristics found in humans [3,30,31]. To mimic human chronic recurrent UC, animals were given multiple low-dose DSS treatments with periods of remission [3].

### 2.2. Body Weight and Diet Intake

Body weights were recorded (Mettler Toledo precision analytical balance) at baseline (d 0), prior to initiating DSS treatment (d 17, 19), prior to the second DSS treatment (d 33), following the second DSS treatment (d 38), prior to the final DSS treatment (d 47), and prior to termination (d 59, 61) [29]. Body weights were used to assess weight loss attributed to the onset or progression of the disease. Intake was measured prior to initiating DSS treatment (d 17), following the second DSS treatment (d 38), and prior to termination (d 59).

### 2.3. Fecal Output and Moisture Content

Feces collected at seven time points corresponding to before and after each DSS treatment, and prior to termination were monitored for consistency and presence of blood. Fresh samples were collected within 15 min of defecation over a 12 h period for two consecutive days, snap-frozen in liquid nitrogen, and stored at −80 °C until further analysis. Daily fecal production was determined using samples collected over 24 h, which were weighed, snap-frozen, and stored at −80 °C until the moisture content was determined [32].

### 2.4. Short-Chain Fatty Acid Analysis

SCFA concentrations in fecal samples collected before and after the first and third DSS treatments and prior to termination were quantified. Samples (~0.30 g) were powdered and extracted before analysis using liquid–gas chromatography [29,32]. To each diluted supernatant (100 μL supernatant, 100 μL 70% ethanol), 200 μL internal standard solution (heptanoic acid in 70% ethanol, Sigma Aldrich, St. Louis, MO, USA) and 20 μL H_3_PO_4_ were added before injection into a Varian 3900 GC (Walnut Creek, CA, USA) fitted with an HP-FFAP 30 m, 0.53 mm i.d. capillary column (Agilent, Santa Clara, CA, USA). Mixed standards (Sigma Aldrich, St. Louis, MO, USA) were used to calculate SCFA concentrations, which were multiplied by the 24-h fecal weight to estimate 24-h SCFA excretion.

### 2.5. Tissue Fixation

After the removal of fecal material, two 1-cm sections were removed from the distal colon, rinsed in RNase-free phosphate buffered saline, and either fixed in 4% paraformaldehyde or 70% ethanol prior to paraffin embedding [17,33].

### 2.6. Injury and Inflammation Scores

Sections of the tissues were stained with H&E and scored for injury and inflammation by a veterinary pathologist without knowledge of the treatment conditions. The extent of epithelial injury and inflammation (score of 0–3) were characterized as previously reported [34,35].

### 2.7. Proliferative Index, Zone, and Crypt Height

From the distal colon, an additional 1 cm was removed and fixed in 70% ethanol (EtOH). Tissues were pre-treated with Reveal Decloaker (Biocare Medical), then stained with Vectastain^®^ Elite^®^ ABC Kit (Vector Lab), and purified proliferating cell nuclear antigen (PCNA) monoclonal antibody (Anti-PC-10, Covance, Emeryville, CA, USA). To visualize positively stained cells, slides were then stained with 3,3′-diaminobenzadine (DAB) Substrate Kit for Peroxidase (Vector Labs, Newark, CA, USA) and counterstained with hematoxylin solution (Sigma Aldrich). Twenty-five crypt columns per animal were observed to calculate crypt column height, proliferative index, and proliferative zone [33,36].

### 2.8. Mucosal Samples

The remaining colon was removed and rinsed twice with RNase-free PBS, and the mucosa was collected by scraping the tissue on a chilled RNase-free surface. One aliquot was homogenized with 500 µL of denaturation solution (Ambion, Austin, TX, USA) in an RNase-free tube prior to storage at −80 °C. The remaining material was homogenized in 400 µL of protein buffer prior to centrifugation (15,000× *g*, 30 min at 40 °C), and the resulting supernatant was removed and stored at −80 °C [29].

### 2.9. NF-κB Activity

NF-κB activity was determined using the manufacturer’s protocol for whole cell lysates with a TransAM^®^ NF-κB Chemi p65 kit (Active Motif, Carlsbad, CA, USA) [19]. NF-κB activity (p50/p65 subunits) is reported in relative luminescence units, which is the level of NF-κB activation in the samples compared to the positive control (Jurkat nuclear extract) provided in the kit [29]. Activity was normalized to protein concentrations, which were quantified using the Coomassie Plus (Bradford) Assay Kit (Thermo Scientific, Waltham, MA, USA).

### 2.10. Real Time RT-PCR

An aliquot of the mucosal scrapings was homogenized on ice in denaturation solution and the mRNA samples were stored at −80 °C until further analysis [17]. Total mRNA was isolated with Phase Lock Gel tubes (5 Prime, Gaithersburg, MD, USA) and a ToTALLY RNA™ Kit (Ambion, Austin, TX, USA) before treatment with DNase (DNA-free Kit, Ambion) [29]. The quality of the isolated mRNA was measured using an Agilent Bioanalyzer (Santa Clara, CA, USA), and mRNA concentrations were measured using spectrophotometry. Superscript™ III Reverse Transcriptase Kit (Invitrogen, Carlsbad, CA, USA), Taqman^®^ Array Microfluidic Cards (Applied Biosystems, Foster City, CA, USA) were used for real-time RT-PCR to reduce the impact of loading differences across target analyses [29]. Then, using an ABI 7900 HT thermocycler (Applied Biosystems), and SDS 2.4 software (Applied Biosystems) expression was quantified for select gene targets (*TFF-3*, *TGF-Β*, *TLR-2*, *TLR-4*, *TLR-5*, *TLR-9*, *TNF-α*, *TNFR-1*, *TNFR-2*, *Tollip*, *FGF-2*, *IκΒα*, *IL-1*, *IL-6*, *IL-12*, *MyD88*, *COX-2*, *RelA/p65*, *RIP kinase*, *SLC5A8*, and *MCT-1*). These targets allowed for the analysis of several pathways that contribute to the regulation of processes involved in maintaining colon health. To complement NF-κB activity data, *RelA/p65* and *IκΒα* were measured, in addition to the pro-inflammatory cytokines downstream of NF-κB (*IL-1*, *IL-6*, *IL-12*, *TNF-α*, *COX-2*, *TGF-B*). Upstream of NF-κB are the TLR (*TLR-2*, *TLR-4*, *TLR-5*, *TLR-9*, *MyD88*, *Tollip*) and TNFR (*TNFR-1*, *TNFR-2*, *RIP kinase*, *TNF-α*) pathways, both of which can function to regulate activation of NF-κB. In addition, injury repair molecules *TFF-3* and *FGF-2* were measured, since DSS is an injury model in addition to an inflammation model. Lastly, the SCFA transport proteins *MCT-1* and *SLC5A8* were measured to complement the SCFA concentrations measured in the feces. The data were analyzed using the comparative C_T_ (ΔΔ C_T_) method in RQ Manager 1.2.2 (Applied Biosystems, Carlsbad, CA, USA), with 18S as the calibrator gene.

### 2.11. Statistics

The data were analyzed using a two-way ANOVA (diet, treatment, diet*treatment) using SAS 9.3 software (SAS Institute, Inc., Cary, NC, USA). All data are reported as least squares (LS) means ± standard error of the mean (SEM), using a significance of *p* < 0.05.

## 3. Results

### 3.1. Body Weight, Intake, and Fecal Moisture Content

There were no differences in body weight between groups for the duration of the experiment, and there were no differences in intake prior to initiating DSS treatments or prior to termination. However, on day 38 of the experiment, non-DSS-treated animals consuming the quercetin diet had lower food intake (17.6 ± 0.8) than non-DSS-treated animals consuming the basal diet (20.04 ± 0.8, *p* = 0.01) and DSS-treated animals consuming the quercetin diet (20.07 ± 0.7, *p* = 0.02).

**Figure 1:**
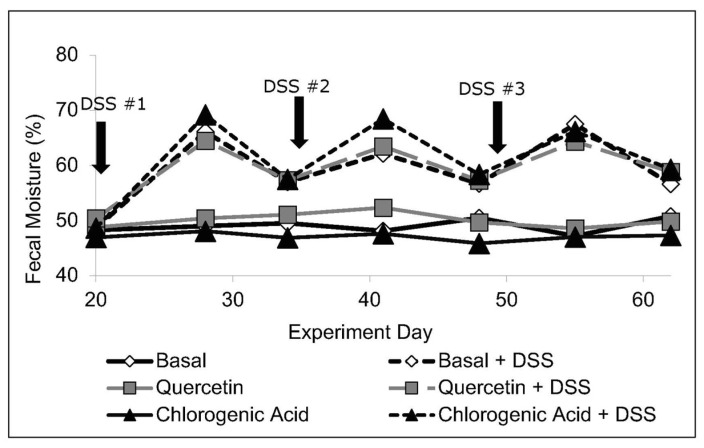
Baseline measurements (day 20, pre-DSS 1) revealed no diet-related differences in fecal moisture content (*n* = 8–11 rats/diet). DSS increased fecal moisture content (*p* < 0.05) and it remained higher than in control animals for the remainder of the experiment.

Although the severity of the disease was not reflected in body weight or intake, fecal moisture content increased with DSS treatment (*p* < 0.05) and remained elevated compared to non-DSS-treated animals (*p* < 0.05). Prior to termination, DSS animals continued to show symptoms of disease, including increased fecal moisture content (*p* < 0.05) (Figure 1).

### 3.2. SCFA Concentrations

**Figure 2:**
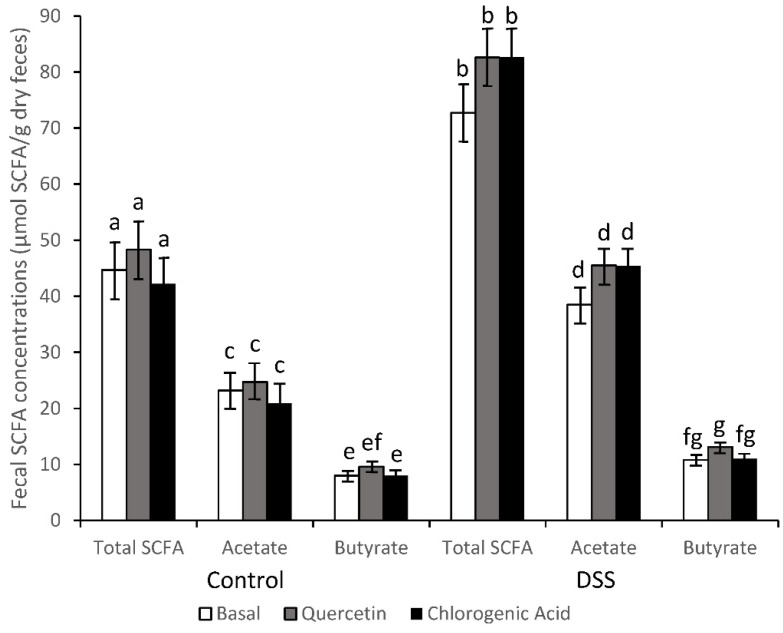
SCFA concentrations remained higher in DSS-treated animals compared to control animals after recovering from the third DSS treatment (acetic acid, *p* < 0.05; butyric acid, *p* < 0.05; total SCFA, *p* < 0.05; *n* = 8–11 rats/diet). Means not sharing a common superscript (Total SCFA, a,b; Acetate, c,d; Butyrate, e,f,g) are significantly different (*p* < 0.05). Data are LS means ± SEM, *n* = 8–11 rats/diet.

Due to the diarrhea observed in DSS-treated animals, SCFA concentrations were corrected for percent water in the feces and reported on a dry weight basis. Prior to DSS treatment, there were no differences in total SCFA concentrations from rats consuming basal, quercetin, or chlorogenic acid diets (53.1, 53.6, and 55.2 µmol/g feces, respectively). Following the first DSS treatment SCFA concentrations were elevated (*p* < 0.05) for all three diet groups (115.6, 89.7, and 115.2 µmol/g feces from rats consuming the basal, quercetin or chlorogenic acid diet). The impact of DSS was apparent after each treatment period. For example, following the final DSS treatment, all animals treated with DSS had higher fecal concentrations of SCFA (acetic acid, *p* < 0.0001; butyric acid, *p* < 0.05; total SCFA, *p* < 0.0001; Supplemental Table S2). Even two weeks after the last DSS treatment, all DSS-treated animals continued to have higher levels of SCFA (acetic acid, *p* < 0.05; butyric acid, *p* < 0.05; total SCFA, *p* < 0.05) compared to their non-DSS counterparts (Figure 2).

Prior to DSS treatments there were no diet effects on relative concentrations of acetic and butyric acids; however, after the final DSS exposure, differences were observed between the DSS-treated rats and their non-DSS counterparts. DSS increased relative concentrations of acetic acid (*p* < 0.05) and decreased relative concentrations of butyric acid (*p* < 0.05) (Supplemental Table S2). After 2 wks of recovery following the final DSS treatment, the animals fed a basal diet had comparable relative concentrations of acetic acid to DSS and non-DSS-treated rats, but the percentage of butyrate remained depressed. The relative concentration of acetic acid (*p* < 0.05) and butyric (*p* < 0.05) acids, however, still showed prominent differences between the DSS and non-DSS-treated rats receiving the quercetin and the chlorogenic acid diets (Supplemental Table S2).

**Figure 3:**
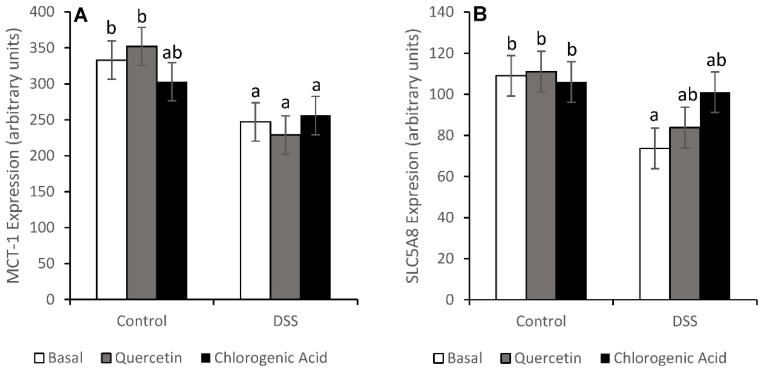
MCT-1 (**A**) and SLC5A8 (**B**) mRNA levels show that DSS reduced the expression of both genes in the basal diet (*p* < 0.05). Animals fed chlorogenic acid were able to mitigate DSS-induced decreases in MCT-1 expression. Both experimental diets were able to mitigate DSS-induced decreases in SLC5A8 expression. Means not sharing a common superscript are significantly different (*p* < 0.05). Data are LS means ± SEM, *n* = 7–8 rats/diet.

Increased concentrations of SCFA in the feces could be caused by an increase in production or a decrease in absorption, so the expression of SCFA transport proteins SLC5A8 and MCT-1 was measured. In animals fed the basal diet, DSS reduced the expression of both SCFA transport proteins (*p* < 0.05). Chlorogenic acid was able to partially mitigate the decrease in MCT-1 expression, while both quercetin and chlorogenic acid mitigated the DSS-induced decrease in SLC5A8 expression (Figure 3).

### 3.3. Injury and Injury Repair

**Figure 4:**
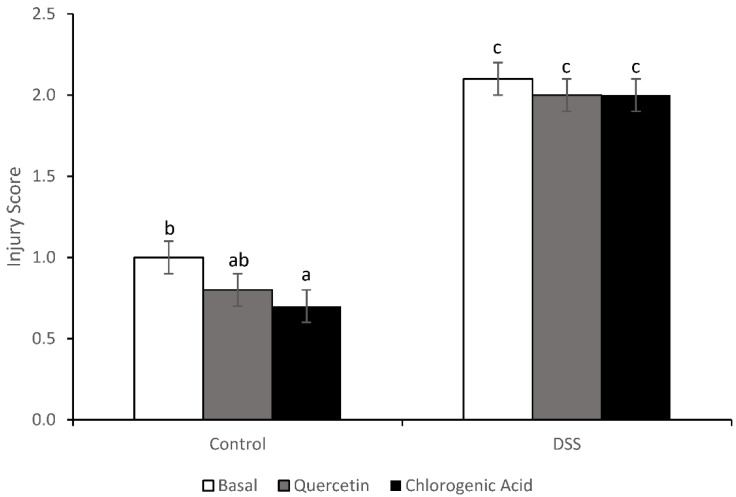
DSS increased the injury score in all diet groups (*p* < 0.0001). In non-DSS-treated animals, chlorogenic acid reduced the injury score compared to the basal diet (*p* = 0.0272). Means not sharing a common superscript are significantly different (*p* < 0.05). Data are LS means ± SEM, *n* = 9–11 rats/diet.

**Figure 5:**
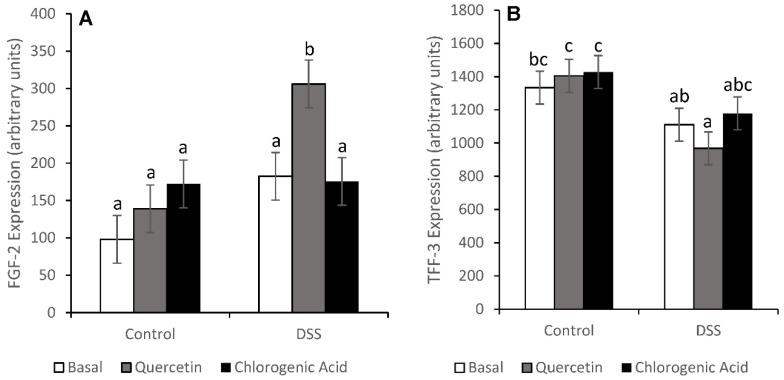
Expression of injury repair molecules suggests that quercetin may be protective by increasing the expression of the basolateral repair molecule FGF-2 (**A**) (*p* = 0.0005). DSS-treated animals fed quercetin had lower levels of TFF-3 expression (**B**), an apical wound healing molecule (*p* = 0.0035). Means not sharing a common superscript are significantly different (*p* < 0.05). Data are LS means ± SEM, *n* = 7–8 rats/diet.

Treatment with DSS increased injury (*p* < 0.0001) scores (Figure 4). Non-DSS-treated animals fed the chlorogenic acid diet had lower injury scores than non-DSS-treated animals fed the basal diet (*p* = 0.0272). Although diet did not have an effect on distal colon injury scores, bioactive compounds did alter the expression of some injury repair molecules. DSS-treated animals fed a quercetin diet had higher expression of fibroblast growth factor-2 (FGF-2) than any other diet/treatment group (*p* < 0.01) (Figure 5). FGF-2 is an injury repair molecule found on the basolateral membrane of epithelial cells. In animals fed the quercetin diet, trefoil factor-3 (TFF-3) expression was lower in DSS-treated animals compared to non-DSS-treated animals (*p* < 0.05). TFF-3 is a wound-healing molecule found on the apical membrane of colonic epithelial cells and is expressed predominantly by goblet cells [37]. A decrease in TFF-3 expression could reflect impaired wound healing on the apical membrane of epithelial cells.

### 3.4. NF-κB Activity and Expression of Downstream Effectors

**Figure 6:**
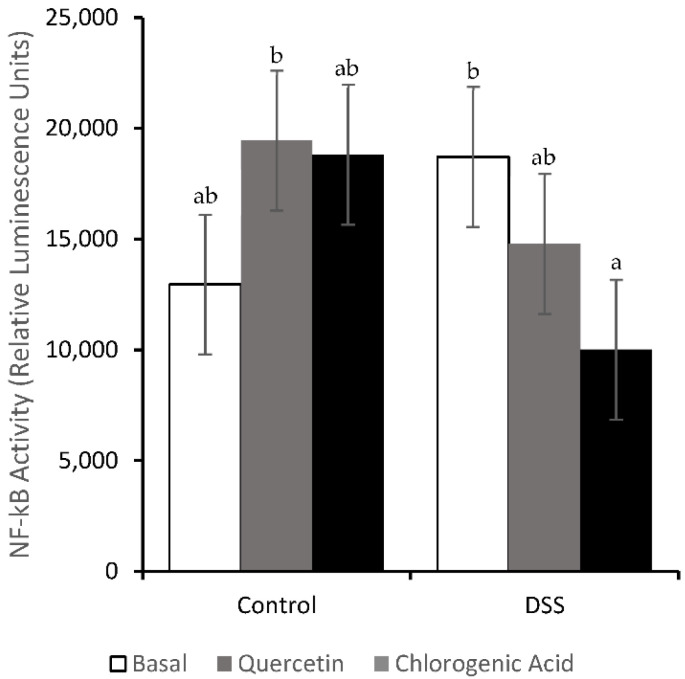
Chlorogenic acid reduced NF-κB activity compared to the basal diet, in DSS-treated animals. Means not sharing a common superscript are significantly different (*p* < 0.05). Data are LS means ± SEM, *n* = 8–11 rats/diet.

**Figure 7:**
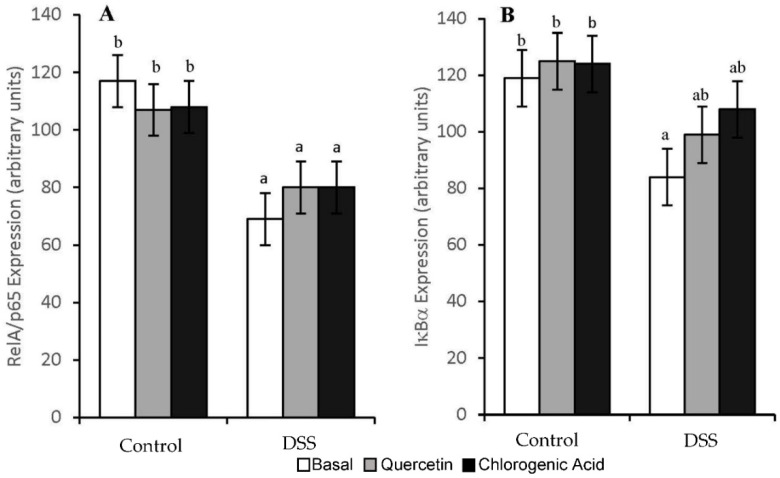
DSS decreased mRNA expression of RelA/p65 (**A**) in all diet groups. Quercetin and chlorogenic acid mitigated the DSS-induced inhibition of IκBα expression observed in rats consuming the basal diet (**B**). Means not sharing a common superscript are significantly different (*p* < 0.05). Data are LS means ± SEM, *n* = 7–8 rats/diet.

DSS increased the inflammation scores (*p* < 0.01) in all diet groups (1.7, 1.6, and 1.6 for Control, Quercetin and Chlorogenic Acid, respectively). However, the activity of NF-κB, a pro-inflammatory transcription factor, was not significantly increased by DSS (Figure 6). DSS-treated animals fed a chlorogenic acid diet had lower levels of NF-κB activity compared to those fed a basal diet (*p* < 0.05). The expression of NF-κB-associated molecules was also measured in mucosal scrapings. In all diet groups, treatment with DSS resulted in lower expression levels of RelA/p65 (*p* < 0.05), the regulatory subunit of NF-κB (Figure 7). In animals fed the basal diet, DSS-treatment also reduced the expression of IκΒα, the molecule that prevents NF-κB translocation into the nucleus (*p* < 0.05). However, both experimental diets were able to partially mitigate the decrease in IκΒα expression induced by DSS.

**Figure 8:**
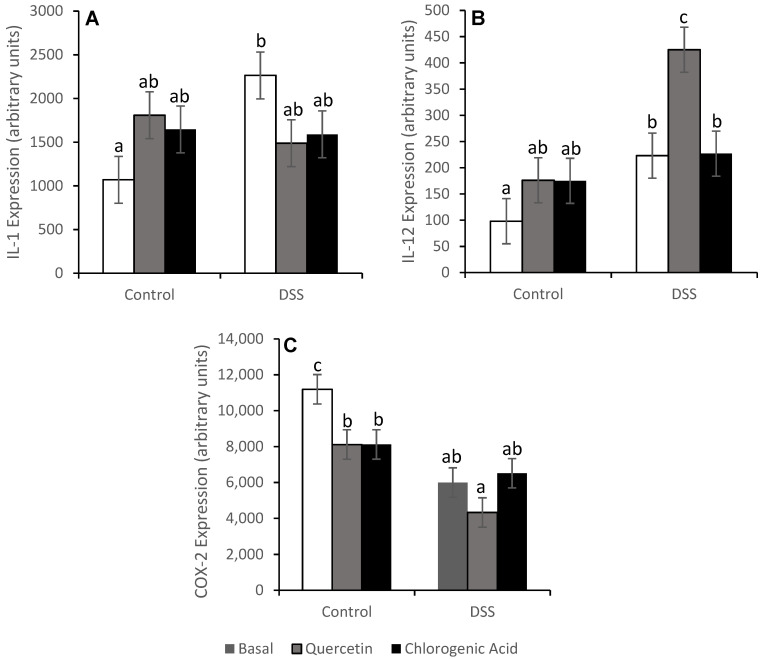
DSS increased the mRNA expression of IL-1 (**A**) and IL-12 (**B**) and reduced the expression of COX-2 (**C**) in animals fed the basal diet. Means not sharing a common superscript are significantly different (*p* < 0.05). Data are LS means ± SEM, *n* = 7–8 rats/diet.

The expression of pro-inflammatory cytokines downstream of NF-κB was also measured (Figure 8). Contrary to what we expected, COX-2 expression in animals fed the basal and quercetin diets and treated with DSS was reduced (*p* < 0.01) relative to the rats consuming those diets but not treated with DSS. Rats fed the basal diet had higher levels of IL-1 (*p* < 0.01) and IL-12 (*p* < 0.05) expression with DSS treatment. The expression of COX-2, IL-1, and IL-12 were not impacted by DSS treatment for animals fed the chlorogenic acid diet. IL-1 expression was also not impacted with DSS treatment for animals fed the quercetin diet, but DSS treatment increased IL-12 expression in these rats (*p* < 0.01). TNF-α, IL-6, and TGF-Β expression were not affected by either diet or DSS treatment (Table 1).

### 3.5. Toll like Receptor Pathways

DSS reduced the expression of TLR-4 in animals fed the basal diet but did not significantly alter the expression of TLR-2, TLR-9, and TLR-5 (Table 2). Animals fed the chlorogenic acid diet had decreased expression of TLR-9 (*p* < 0.01) with DSS-treatment, a potentially protective TLR. Another protective TLR, TLR-2, had increased expression with quercetin supplementation in non-DSS-treated rats compared to those consuming the basal diet (*p* < 0.05).

All TLRs measured can activate NF-κB through the adaptor protein MyD88. Treatment with DSS reduced MyD88 expression in rats consuming the basal and quercetin diets (*p* < 0.05) (Table 2). Tollip inhibits MyD88-induced activation of NF-κB, and Tollip expression was reduced by DSS treatment in rats fed the basal diet (*p* < 0.05) (Table 2). Chlorogenic acid maintained MyD88 expression levels with DSS treatment, while both quercetin and chlorogenic acid maintained Tollip expression levels.

### 3.6. Tumor Necrosis Factor Receptor Pathways

**Figure 9:**
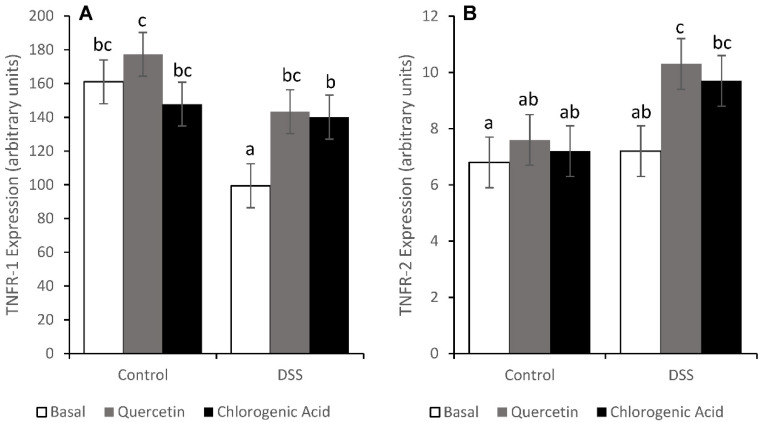
DSS reduced the mRNA expression of TNFR-1 (**A**) in animals fed a basal diet. Quercetin and chlorogenic acid maintained the expression of TNFR-1. The level of TNFR-2 (**B**) mRNA was elevated in DSS-treated animals on the quercetin diet. Means not sharing a common superscript are significantly different (*p* < 0.05). Data are LS means ± SEM, *n* = 7–8 rats/diet.

Although TNF-α expression was not affected by diet or treatment, TNFR expression was altered. The expression of TNFR-1, the pro-apoptotic TNFR, was reduced by DSS treatment in rats fed the basal diet (*p* < 0.05) (Figure 9). However, rats fed the quercetin and chlorogenic acid diets had comparable levels of TNFR-1 between DSS and non-DSS-treated animals. In DSS-treated animals, RIP kinase (RIPk) expression was comparable across diets (34.9, 35.3, and 38.5 for Control, Quercetin and Chlorogenic Acid, respectively). Downstream effects of RIP include NF-κB activation and apoptosis via the activation of caspase-8, -10, and -3. The expression of TNFR-2, which promotes cell survival and proliferation, was increased by the quercetin diet in DSS-treated animals (*p* < 0.05).

### 3.7. Proliferation in the Distal Colon

No significant differences were observed with DSS treatment for the proliferative zone (PZ), proliferative index (PI), or crypt height (CH) (Table 3). DSS-treated animals fed the chlorogenic acid diet had shorter crypt heights compared to DSS-treated animals receiving the basal diet.

## 4. Discussion

Diet-based interventions capable of mitigating the inflammatory sequelae occurring in the colon are needed because IBD remains an incurable condition [38]. Plums have been shown to have anti-cancer (dried plums) and anti-inflammatory (plum phenolic extract) properties [15,16]. Quercetin and chlorogenic acid, found in stone fruits in relatively high concentrations, have demonstrated anti-inflammatory properties in acute models of colitis in which animals consumed diets containing non-fermentable fibers [19,21], yet the ability of these bioactive compounds to combat chronic ulcerative colitis when animals are consuming fibers representing those predominantly found in stone fruits has not been investigated. We used a chronic, recurrent colitis model to investigate the damaging effects of DSS and the possible protective mechanisms of quercetin and chlorogenic acid.

Changes in colonic microbiota toward more pathogenic bacteria or decreased production of beneficial molecules, like butyrate, could increase inflammation, and altered fecal concentrations of SCFA, including butyrate, have been reported in UC [3,5,39]. Dietary fiber influences microbial populations and their metabolites, and since chlorogenic acid and quercetin are also metabolized by the microbiota, it is possible these bioactive compounds may have different impacts on UC when pectin is the fiber source, as opposed to a non- or lightly-fermented fiber source [22,24,25,26]. We observed an increase in fecal SCFA concentrations with DSS treatment, which could be the result of either changes in the microbiota, altered SCFA uptake into colonocytes, or both. Although we did not measure butyrate uptake or oxidation, slight decreases in butyrate concentrations during recovery phases (compared to the active disease state) could indicate an enhanced uptake and/or utilization compared to the active disease state. Quercetin could play a role in maintaining the flux of butyrate; following the first DSS treatment, butyrate levels were comparable between DSS and non-DSS-treated rats consuming the quercetin diet. Considering that very high concentrations of butyrate may be toxic during active ulcerative colitis [40], quercetin may be able to mitigate some of the damage observed with acute colitis. However, this effect was not observed later in the experiment, possibly due to the extensive damage caused by the multiple exposures to DSS. The increased levels of fecal SCFA suggest that fermentation was not impaired by DSS treatment, but we did observe changes in the relative concentrations of butyrate (decrease) and acetate (increase) with DSS treatment, further suggesting there were changes in microbial populations, fermentation patterns, or substrate uptake into epithelial cells. Future work should analyze fecal samples from the proximal and distal colon collected at termination to determine if fermentation rates and the microbiome are affected by colitis and by these dietary interventions.

The high fecal butyrate concentrations in feces after DSS could be due to a decreased expression of MCT-1 and butyrate uptake, which has already been demonstrated in DSS-induced acute colitis [41]. If butyrate absorption was limited by DSS treatment, it would lead to altered metabolism by colonocytes [42]. Mitigation of DSS-induced decreases in MCT-1 expression by chlorogenic acid and in SLC5A8 expression by both quercetin and chlorogenic acid suggests that uptake may not have been impaired; however it would be necessary to document tissue levels of these proteins to determine if actual capacity for transport was retained.

Butyrate is thought to have anti-inflammatory and anti-cancer properties [39]. The increased concentrations of fecal butyrate could reflect high concentrations in the lumen; however, diet had no effect on the injury or inflammation score. Likewise, both quercetin and chlorogenic acid derivatives have been shown to have anti-inflammatory properties, but no change was observed in the injury or inflammation score in our study [19,21]. The injury caused by DSS was severe, possibly too severe for quercetin and chlorogenic acid to show protection on a phenotypic level. In one acute colitis experiment in which hydrocaffeic acid was identified as being anti-inflammatory, cellulose was used as the fiber source [21]. While this acute model used a higher DSS dose (4%), the cellulose (a non-fermentable fiber) in the diet may have limited DSS interaction with the epithelium. In contrast, the pectin used in the current study is readily fermented in the proximal colon and would not be able to provide the same level of dilution as cellulose.

However, gene expression data did suggest that quercetin and chlorogenic acid might function to enhance injury repair molecules and suppress inflammation pathways. Quercetin has also been shown to enhance epithelial barrier function in vitro by increasing expression of claudin-4, a tight junction protein [18]. Injury repair molecules can also play an important role in the maintenance of the epithelial barrier in UC. A decrease in TFF-3, an apical wound-healing molecule, was observed with DSS treatment in animals fed a quercetin diet. Since TFF-3 is also an injury repair molecule, this is not considered beneficial. TFF-3 is expressed by goblet cells, suggesting a decrease in the number or function of goblet cells in animals fed quercetin [37]. Although apical injury repair may be impaired in those fed a quercetin diet, quercetin could enhance wound repair by rebuilding the tissue from the basolateral membrane through the enhanced expression of FGF-2. Due to the extensive damage to the epithelium and high levels of fecal moisture content (diarrhea), the number of goblet cells could have decreased, thereby reducing the level of TFF-3 expression. However, due to the extent of the injury caused by DSS, injury repair deeper in the tissues may be more important than apical repair.

Proliferation is also necessary for the maintenance of the epithelial barrier. Previous studies of acute colitis have shown that proliferation was increased during the active state of disease [43]. Hyper-proliferation, in conjunction with inflammation, can lead to tumor formation [44]. Since our animals were terminated during a period of remission, proliferation levels (proliferative index and proliferative zone) were comparable across diets and treatments. If the animals had been terminated during an active state of the disease, changes in proliferation due to diet and/or treatment may have been observed.

These bioactive compounds did affect pathways that regulate the cell cycle. Quercetin and chlorogenic acid mitigated DSS-induced decreases in TNFR-1 expression. In addition, quercetin reduced the expression of TNFR-2 in DSS-treated animals. Through RIP and caspase-8, TNFR-1 is able to induce cell death [45]. TNFR-2 also plays a role in the cell cycle during inflammation, although it is responsible for cell proliferation and survival, rather than cell death [46]. TNFR mRNA expression data would suggest that DSS-treated animals consuming a quercetin diet would have decreased levels of proliferation, although these changes were not observed. Despite the changes in TNFR expression, we cannot conclusively discuss downstream changes. TNFR must be stimulated by TNF-α or other ligands to trigger downstream biological responses [45]. Our study showed no increase in TNF-α expression with diet or DSS treatment, which could be the reason why no change was observed in proliferation rates. By maintaining levels of TNFR-1 gene expression and increasing TNFR-2 expression in DSS-treated animals, chlorogenic acid and quercetin could impact the cell cycle.

Both TNFR-1 and -2 also function to activate NF-κB, which is not only anti-apoptotic, but also promotes the expression of pro-inflammatory cytokines [46]. NF-κB is also activated through TLR pathways and MyD88 transduction molecules [47,48]. Chlorogenic acid decreased NF-κB activation in DSS-treated animals when compared to those on the basal diet. When evaluating the effect of chlorogenic acid on NF-κB-related expression, we observed the maintenance of IκΒα, MyD88, and Tollip expression. Quercetin mitigated DSS-induced decreases in Tollip and IκΒ expression. Since dysregulation in NF-κB activation occurs in UC, chlorogenic acid and quercetin may be able to maintain the regulation of NF-κB activation by suppressing DSS-induced changes in NF-κB regulatory molecules. The proper regulation of NF-κB in epithelial cells could be protective in colitis [5,7,44].

Due to the high level of inflammation in chronic colitis, an elevation in NF-κB was expected. The elevation in NF-κB activity in the basal diet with DSS treatment was not significant, but the expression of NF-κB-associated molecules (RelA/p65 and IκΒα) was reduced by DSS treatment. The reduced expression of NF-κB could be why NF-κB activation was not elevated as we expected. Downstream of NF-κB, the expression of pro-inflammatory cytokines was affected by both diet and treatment. DSS-treated animals on the basal diet showed increases in IL-1 and IL-12. Quercetin has been shown to inhibit inflammation in acute DSS-induced colitis by suppressing NF-κB activation [19]. Quercetin was able to mitigate DSS-induced increases in IL-1, a severe pro-inflammatory cytokine and activator of NF-κB. Hydrocaffeic acid has also been shown to alleviate DSS-induced inflammation in acute colitis by suppressing the expression of pro-inflammatory cytokines [21]. In our study, the expression levels of COX-2, IL-1, and IL-12 were comparable between the non-DSS and DSS-treated animals on the chlorogenic acid diet. Quercetin and chlorogenic acid may function through TLR and NF-κB pathways to influence the expression of pro-inflammatory cytokines.

Although elevated COX-2 levels were expected with DSS treatment, reduced levels of COX-2 were actually observed. COX-2 can promote inflammation and tumorigenesis but can also be protective when expressed in epithelial cells [44]. COX-2 may contribute to the decreased paracellular permeability to bacterial translocation into the lamina propria [44]. The reduced expression of COX-2 with DSS treatment in animals fed basal and quercetin diets could be harmful, especially since dysbiosis and impaired integrity of the epithelium occurs in UC. Quercetin did reduce COX-2 expression in non-DSS-treated animals, as occurred in a prior experiment using the AOM model of colon cancer [17].

The ability of quercetin and chlorogenic acid to downregulate the expression of pro-inflammatory cytokines may not be the only mechanism of protection in UC. Altering the luminal environment (fecal SCFA) and mRNA expression of SCFA transporters, in addition to enhancing injury repair molecule mRNA expression could protect the epithelium in colitis. Although the injury and inflammation scores in this study did not show protection by quercetin and chlorogenic acid, their influence on mRNA expression of molecules in TLR, TNFR, NF-κB, and wound healing pathways suggests a multifaceted protection in colitis.

One limitation of this study is the use of a chemically induced animal model of ulcerative colitis. Although it allows a high degree of control over the intake of a strictly defined diet, it cannot replicate the human condition. Another limitation is that the experimental diets do not reflect the consumption of intact foods by humans. A distinct advantage of this study is that since quercetin and chlorogenic acid are found together with the fermentable fiber pectin in fruits and vegetables, the responses reported in this paper may better reflect the reactions to consuming intact stone fruits than in prior works. However, further investigations are necessary to identify any additive or synergistic effects obtained when these bioactive compounds are combined.

## Figures and Tables

**Table 1:** Expression (arbitrary units) of pro-inflammatory cytokines downstream of NF-κB in the scraped colon mucosa of rats ^1^.

	Basal Diet		Quercetin Diet		Chlorogenic Acid Diet	
	Control	DSS	Control	DSS	Control	DSS
IL-6	752 ± 168 ^a^	544 ± 168 ^a^	923 ± 168 ^ab^	1365 ± 180 ^b^	828 ± 168 ^a^	606 ± 168 ^a^
TGF-Β	152 ± 18 ^a^	147 ± 18 ^a^	179 ± 18 ^a^	177 ± 20 ^a^	161 ± 18 ^a^	170 ± 18 ^a^
TNF-α	1804 ± 354 ^a^	2090 ± 354 ^ab^	2596 ± 354 ^ab^	2991 ± 378 ^b^	2543 ± 354 ^ab^	1662 ± 354 ^a^

^1^ Values are LS means ± SEM. Means not sharing a common superscript differ (*p* < 0.05). *n* = 7–8 rats/group.

**Table 2:** Expression (arbitrary units) of TLRs, MyD88, and Tollip in scraped colon mucosa of rats ^1^.

	Basal Diet		Quercetin Diet		Chlorogenic Acid Diet	
	Control	DSS	Control	DSS	Control	DSS
TLR-2	3.9 ± 1.0 ^a^	6.6 ± 1.0 ^ab^	6.9 ± 1.0 ^b^	7.7 ± 1.7 ^b^	6.2 ± 1.0 ^ab^	7.7 ± 1.0 ^b^
TLR-4	61.4 ± 5.5 ^b^	30.3 ± 5.5 ^a^	55.0 ± 5.5 ^b^	34.9 ± 5.9 ^a^	55.7 ± 5.5 ^b^	36.5 ± 5.5 ^a^
TLR-5	30.1 ± 3.6 ^ab^	25.3 ± 3.6 ^ab^	31.2 ± 3.6 ^ab^	22.7 ± 3.8 ^a^	35.1 ± 3.6 ^b^	26.1 ± 3.6 ^ab^
TLR-9	7.0 ± 1.2 ^abc^	4.7 ± 1.2 ^ab^	7.6 ± 1.2 ^bc^	7.9 ± 1.3 ^bc^	8.9 ± 1.2 ^c^	3.9 ± 1.2 ^a^
MyD88	99.7 ± 8.4 ^c^	60.4 ± 8.4 ^a^	104.8 ± 8.4 ^c^	70.5 ± 8.9 ^ab^	91.8 ± 8.6 ^bc^	74.2 ± 8.4 ^ab^
Tollip	58.1 ± 4.2 ^c^	42.7 ± 4.2 ^a^	57.0 ± 4.2 ^bc^	45.1 ± 4.4 ^ab^	56.7 ± 4.2 ^bc^	46.8 ± 4.2 ^abc^

^1^ Values are LS means ± SEM. Means not sharing a common superscript differ (*p* < 0.05). *n* = 7–8 rats/group.

**Table 3:** Colonocyte proliferation in the distal colon ^1^.

	Basal Diet		Quercetin Diet		Chlorogenic Acid Diet	
	Control	DSS	Control	DSS	Control	DSS
CH ^2^	22.8 ± 1.0 ^ab^	24.8 ± 1.0 ^b^	20.9 ± 1.0 ^a^	22.2 ± 1.1 ^ab^	21.3 ± 1.0 ^a^	21.9 ± 1.0 ^a^
PI ^2^	16.6 ± 2.4 ^ab^	15.0 ± 2.4 ^a^	20.0 ± 2.4 ^ab^	20.0 ± 2.7 ^ab^	22.2 ± 2.4 ^b^	18.0 ± 2.4 ^ab^
PZ ^2^	39.0 ± 2.5 ^a^	37.5 ± 2.5 ^a^	41.3 ± 2.5 ^a^	39.3 ± 2.8 ^a^	43.8 ± 2.5 ^a^	40.6 ± 2.5 ^a^

^1^ Values are LS means ± SEM. Means not sharing a common superscript differ (*p* < 0.05). *n* = 8–10 rats/group. ^2^ Abbreviations: crypt height (CH, number of cells/crypt column), proliferative index (PI, percentage of cells in a crypt column undergoing proliferation), proliferative zone (PZ, position of the highest proliferating cell in a crypt column divided by the total number of cells in a crypt column).

## Data Availability

The data generated from this research are available from the corresponding author.

## Supplementary Materials

The following supporting information can be downloaded at: https://www.mdpi.com/article/10.3390/nu14183706/s1, Table S1: Composition of the basal and experimental diets; Table S2: Fecal SCFA of control or DSS-treated rats consuming a basal diet or diets containing quercetin or chlorogenic acid.
