# Supplementary material for: Chlorogenic Acid and Quercetin in a Diet with Fermentable Fiber Influence Multiple Processes Involved in DSS-Induced Ulcerative Colitis but Do Not Reduce Injury
Source: Nutrients. 2022 Sep 8;14(18):3706. doi: 10.3390/nu14183706 (PMC9501002; doi:10.3390/nu14183706)
Supplement: Supplementary file 1 [file nutrients-14-03706-s001.zip › Supplementary_Material.pdf]

Supplementary Material

Supplemental Table S1. Composition of the basal and experimental diets provided to rats before and during DSS treatment to induce ulcerative colitis

Ingredient	Basal	Quercetin	Chlorogenic Acid
		%	
Dextrose[1]	51.06	50.61	51.01
Casein[1]	22.35	22.35	22.35
DL-methionine[1]	0.34	0.34	0.34
Mineral mix[1]	3.91	3.91	3.91
Vitamin mix[1]	1.12	1.12	1.12
Choline bitartrate[1]	0.22	0.22	0.22
Pectin[2]	6.00	6.00	6.00
Corn oil[3]	15.00	15.00	15.00
Quercetin[4]	-	0.45	-
Chlorogenic acid[4]	-	-	0.05

[1] Harlan, Madison, WI

[2]Gum Tech, Tuscon, AZ

[3]DYETS, Bethlehem, PA

[4]Sigma, St. Louis, MO

Supplemental Table S2. Fecal SCFA of control or DSS-treated rats consuming a basal diet or diets containing quercetin or chlorogenic acid[1]

	Basal Diet		Quercetin Diet		Chlorogenic Acid Diet	
SCFA	Control	DSS	Control	DSS	Control	DSS
			(µmol/g dry weight feces)			
Post DSS-3						
Acetic	24.9±5.1[a]	73.4±4.8[c]	22.4±5.1[a]	53.4±4.6[b]	23.3±4.8[a]	74.0±4.8[c]
Butyric	7.9±1.7[a]	17.7±1.6[b]	8.9±1.7[a]	13.9±1.5[b]	8.3±1.6[a]	15.1±1.6[b]
Total SCFA	47.1±7.7[a]	120.4±7.3[c]	44.5±7.7[a]	90.8±6.9[b]	45.8±7.3[a]	118.1±7.3[c]
Final recovery period						
Acetic	23.2±3.6[a]	38.5±3.2[b]	24.7±3.6[a]	45.5±3.4[b]	20.9±3.8[a]	45.4±3.2[b]
Butyric	8.0±0.9[a]	10.8±0.8[bc]	9.6±0.9[ab]	13.1±0.8[c]	8.0±0.9[a]	11.0±0.8[bc]
Total SCFA	44.7±6.1[a]	72.7±5.5[b]	48.3±6.1[a]	82.6±5.8[b]	42.2±6.5[a]	82.7±5.5[b]
			%			
Post DSS-3						
Acetic	52.3±1.5[a]	60.7±1.4[bc]	50.5±1.5[a]	57.6±1.4[b]	50.8±1.4[a]	62.6±1.4[c]
Butyric	17.4±1.0[bc]	14.3±1.0[a]	19.9±1.0[c]	15.3±1.0[ab]	18.2±1.0[c]	12.8±1.0[a]
Final recovery period						
Acetic	51.5±1.2[ab]	52.6±1.1[abc]	50.5±1.2[a]	55.1±1.2[bc]	49.6±1.3[a]	54.7±1.1[c]
Butyric	18.2±0.8[a]	14.9±0.8[b]	20.2±0.8[a]	15.9±0.8[b]	18.9±0.8[a]	13.7±0.8[b]

[1] Values are LS means ± SEM. Means without a common superscript differ ($p<0.05$). n=8-11 rats/group.
